# A case study from a senior care navigator program: helping older adults address health and social care needs

**DOI:** 10.3389/fpubh.2026.1771025

**Published:** 2026-03-19

**Authors:** Paige Coyne, Philesha Gough, Maria Santana-Garcés, Laura Susick, Lonni Schultz, Shetoya Rice, Nubia Brewster, Maya Zreik, Rob Behrendt, Veronica Bilicki, Sara Santarossa

**Affiliations:** 1 Department of Public Health Sciences, Henry Ford Health, Detroit, MI, United States; 2 Henry Ford Health + Michigan State University Health Sciences, East Lansing, MI, United States; 3 Department of Care Experience, Henry Ford Health, Detroit, MI, United States; 4 College of Human Medicine, Michigan State University, East Lansing, MI, United States

**Keywords:** advocacy, coordination, patient navigation, social determinants of health, support

## Abstract

The population in the United States is rapidly aging. Older adults commonly report complex care needs and are at increased risk for chronic disease, illness, and disability. However, navigating health and social care systems to address care needs can be challenging due to their siloed nature. This intervention’s objective is to assist older adults in navigating health and social care systems and addressing their care needs. Henry Ford Health has implemented a Senior Care Navigator Program (SCNP) through which aging patients receive support coordinating their care, accessing relevant information and resources, and advocacy support to ensure their voice is heard. Since its inception in June 2021 through December 2024, the SCNP and its senior navigators have supported more than 250 patients with their health and social care, providing evidence of its impact. Moreover, increasing need and interest for the SCNP has resulted in its growth, namely through the addition of a second senior navigator and its expansion from 2 clinics to 5 clinics over a 4-year period. Further evidence of the SCNP’s impact can be observed through patient stories, one of which is presented in this paper. The program described in this paper shows potential for helping seniors navigate their care. Senior navigators can support patients by arranging community resources, providing emotional support, and improving patient satisfaction when challenged by complex social or healthcare needs. Expansion of similar programs and efforts will become necessary as the needs of the aging U.S. population continue to grow over time.

## Introduction

Driven in large part by the baby boomer generation, the United States population is aging at an unprecedented rate. By 2034, older adults are projected to outnumber children for the first time in the nation’s history (1). As the proportion of individuals aged 65 years and older continues to rise, so does their need for health and social (i.e., social determinants of health) care. At higher risk for multiple health problems, chronic illness, and disability (2, 3), older adults often have complex care needs. To adequately meet such needs, they must navigate intricate health and social care systems. Unfortunately, navigating these systems can be extremely challenging and can result in less than desirable care if not helmed properly. Difficulties with managing healthcare is caused by numerous factors, including but not limited to a lack of informal caregivers for older adults. Previously, it was common for family members, such as a spouse or an adult child, to act as informal caregivers for these aging adults, including assisting them in navigating health and social care systems in order to meet their care needs. However, with declines in marriages, increases in divorce, and lower fertility, more older adults are without a family member to rely on for assistance.

Patient navigation programs (PNPs) have emerged as a means of supporting older adults in navigating health and social care systems, enabling them to overcome barriers and obtain the care they require (4). To help bridge health and social care gaps and empower older adult patients to stay in their preferred residence for as long as possible, Henry Ford Health (HFH) has created a Senior Care Navigator Program (SCNP) through which aging patients receive support coordinating their care, accessing relevant information and resources, and learning self-advocacy in clinical settings. In this paper, we hope to highlight the need for PNP’s like HFH’s SCNP and the impact such programs can have on older adult patients’ health and wellbeing.

## Context

Established through private donor funding, the SCNP aids older adult patients (and their caregivers) struggling to negotiate health and social care systems at HFH and in its surrounding communities, by offering support via navigation, information, advocacy, and access to resources. The SCNP is run by 2 full-time senior navigators (SNs) who establish themselves as the point of contact for all health and social care needs for patients enrolled in the program. SNs are certified community health workers with additional training in advanced care planning who are equipped to carry out various duties, including but not limited to assessment and monitoring, planning and problem solving, education and advocacy, and coaching. For example, SNs can help ensure patients schedule and keep their medical appointments (and come prepared), verify that patients are getting and taking their prescriptions appropriately, and connecting patients with community or government programs to address social needs like housing, food, and transportation.

In addition, SNs take part in an extensive, 160-h onboarding process whereby they familiarize themselves with all HFH’s relevant policies and procedures, receive computer and electronic health record system training, learn about all hospital, community, and government-based resources at their disposal, and have the opportunity to meet relevant clinic and office staff. Moreover, the SCNP’s program manager conducts weekly 1-h check-in meetings with the SNs and assigns additional training as needed.

Patients aged 50 years or older are enrolled into the SCNP on a referral basis. Most patients were female with an average age of 74.9 years. Most often, primary care providers (or a member of the primary care team) at clinics where the SCNP is offered, are asked to identify older adult patients in need of support. The provider then discusses the SCNP with the patient. If the patient would like to participate in the program, the provider/care team member can initiate a direct referral. Alternatively, during in-clinic days, SNs set up a SCNP information table and older adult patients who self-identify as needing care support can initiate a self-referral. Upon receiving a referral, a SN will get in touch with the patient via telephone. During this introductory telephone call, the SN will provide the patient with an overview of the program, confirm their interest in participating, and begin collecting information regarding their current care gaps. The SN will then conduct a home visit and work in tandem with the patient (and the larger care team) to establish and execute a support/care plan, which typically includes regular check-ins with the SN. As detailed elsewhere (5), some patients opt into participating in a formal program evaluation of the SCNP. In brief, the patients who choose to join the evaluation group, verbally consented, and have one-on-one interviews with a research team member at baseline, 3-, 6-, and 9-month post-initial contact to complete 5 patient-reported outcome measures (5).

Ethical approval was received from HFH’s Institutional Review Board (#14864 and #17452), and all procedures were conducted in accordance with approved protocols. This manuscript provides an exemplar of the SCNP’s impact through a patient story, recounted by a SN. Verbal assent was obtained from the patient for their anonymized information and story to be published in this article.

## Outcomes of the intervention

Since introducing the SCNP in June 2021, the program continues to grow and create positive impact. Initially, the SCNP was only offered through 2 clinics and employed a single SN but has since expanded to 8 clinics and 2 SNs to accommodate increasing need and interest. As of June 2025, 251 patients have enrolled in the SCNP, with a smaller subset (*n* = 57, 22%) opting to participate in a formal program evaluation to assess patient reported outcome measures (i.e., mental health, physical health, etc.) (5). Although there are a number of ways in which we have (5) and will continue to quantify the program’s impact, we feel strongly that use cases (stories that show the patient’s experience with a program) (6), like the one presented below, are the best way to showcase the complete and compelling impact the SCNP can have on its users.

### Patient story

The patient, a 79-year-old Black woman, presented to one of HFH’s gerontology inter-medicine clinics for a new patient appointment. During her visit, a member of the primary care team noted that she had several unmet health and social care needs, was having difficulties finding and accessing supportive resources and services, and would benefit greatly from receiving additional support, which prompted a referral to the SCNP. Upon receiving the referral, a SN with a background in faith and community nursing was assigned to work with the patient.

The SN contacted the patient via telephone shortly after she attended her new patient appointment. During that call, the SN provided the patient with information about the SCNP and confirmed her interest in enrolling. The SN also spent time learning about the patient’s complex health and social needs and scheduled a home visit. She suffered from chronic and severe pain in her hips and back, was a fall risk, had trouble sleeping, depression, struggled to communicate her needs with healthcare providers, and was unable to complete basic daily living tasks (e.g., preparing food, bathing, and housekeeping), among other concerns. When asked about familial support, the patient explained that she lived alone, lacked transportation, and had limited support, despite several family members living nearby. The patient was also adamant that she wanted to remain in her current home and did not want to move into an assisted living residence. The SN concluded that, without intervention, it was unlikely that the patient would be able to navigate existing health and social care systems on her own to meet her needs and safely remain in her home. The SN ended their first call by reassuring the patient that she was in good hands and that the SCNP would help get her the care support she needed.

With a better understanding of the patient’s needs and preferences, and in conjunction with the patient and her primary care team, the SN created a plan of action, which started with prioritizing the patient’s most basic needs: food, personal hygiene, and housekeeping. For example, to address the patient’s meal preparation needs, the SN provided her with a telephone number for Meals on Wheels (7), a state and federally funded program that delivers ready-made meals to homebound seniors. Similarly, the SN also worked with the case manager from the patient’s primary care team to identify additional programs that could bridge gaps related to bathing and housekeeping.

During the patient’s home visit, which occurred a few days after their initial telephone consultation, the patient confirmed to the SN that she had called Meals on Wheels (7) and that she was expecting a call back from her local chapter within a week’s time. The SN then shared with the patient that she had followed up with her primary care team and had identified resources and programs to address all her outstanding health and social care needs. Given that the patient’s needs were numerous and widespread, the SN informed the patient that she was eligible for the Program of All-Inclusive Care for the Elderly (PACE) (8), a comprehensive program that helps older adults meet their health and social care needs in the community instead of going to a care facility. The SN explained to the patient that if she enrolled, all of her health and social needs could be serviced through a single program. Open to learning more about the program, the SN connected the patient with a representative from PACE. After ending her call with the PACE representative, the patient told the SN that she was interested, but unsure if she wanted to enroll and wanted to discuss the program with a family member before finalizing her decision. The SN informed the patient that she could take all the time she needed, and that the SN had other information and resources she could share with the patient in the meantime. Specifically, the SN discussed and shared the National Institutes of Health resources (9) for *Getting a Good Night’s Sleep* (10), *Understanding Depression* (11), and *Talking With Your Doctor* (12) with the patient to help her with her sleep, depression, and confidence in talking with healthcare providers. The SN also discussed and demonstrated deep breathing techniques to help the patient relax and manage her pain.

The SN continued to follow up, via telephone, with the patient at regular intervals (weekly, to biweekly, to monthly). During this time, the patient and the SN were able to address most of the patient’s needs, which allowed her to remain independent and in her preferred residence, her home. Notably, although the patient decided not to enroll in PACE, the SN was able to support the patient in obtaining weekly deliveries of premade meals and in-home aid to help with bathing and housekeeping. Using her background and expertise in faith and community nursing, the SN also provided both emotional and spiritual support to the patient via the regular check-in telephone calls, which anecdotally had a positive impact on the patient’s depression. Finally, she was able to help the patient advocate for herself and obtain an appointment with a specialist provider who identified the cause of the patient’s hip and back pain and created a plan to manage her pain. In total, the patient was enrolled in the SCNP for over 3 years.

### Lessons learned

Through the administration of the SCNP, several important lessons have been learned. Specifically, by (a) providing support that does not overstep a patient/caregiver’s agency, (b) ensuring navigational support is personalized, and (c) employing a holistic approach to providing support, SNs can maximize the positive impact the SCNP has on its patients/caregivers.

*Patient/Caregiver agency.* With regards to agency, all health and social care decision making should reflect, and be respectful of, older adult patients’ preferences and goals (13–16). This means that SNs are responsible for supporting older adult patients navigating health and social care systems, but it is not their place to make needs-related decisions on behalf of the patient. Rather, SNs work best when they establish a trusting relationship with their patients and provide them with the necessary information, resources, and advocacy to support independent decision making. For the aforementioned patient, this included respecting her decision to forgo PACE in favor of separate services for providing meals, bathing, and housekeeping. Although this required more work for the SN, it allowed the patient to maintain agency in her decision making. Anecdotally, this allowed her to feel comfortable with the services she received and ensured that she could trust her SN to help her address subsequent needs, while also increasing her satisfaction with the program.

#### Personalized support

For the SCNP to be successful, it is also imperative that SNs provide personalized navigational support (17–19). In doing so, SNs can ensure the support they are providing is tailored to the individualized needs of each patient, allowing the SN and patient to work jointly toward addressing the patient’s health and social care needs. Additionally, this also requires SNs to recognize that different approaches may be required to address the same health or social care needs of different patients. For example, many older adults who enroll in the SCNP struggle to effectively communicate with their providers; however, the approach a SN takes to increase a patient’s efficacy in communicating with a provider may differ. In some cases, it could simply mean sharing a resource that delineates strategies for communicating with a provider. In other cases, like the previously discussed patient’s case, it may require a more intensive approach, whereby resources are shared but the SN also spends additional time teaching the older adult patient how to apply the information during their medical visits or touching base with the patient before provider visits to go over what they want to discuss and what questions they should ask.

#### Holistic approach

Lastly, SNs must take a holistic approach to providing navigational support (16, 20, 21). Providing optimal support entails attending to a patient’s emotional well-being, spiritual health, and social care needs in addition to their physical needs. A holistic approach also emphasizes the need for the SN and patient to work in partnership and have mutual understanding in order to address the patient’s needs most effectively. There is also an understanding that patient needs may change over time. For the aforementioned patient, this required the SN to understand that the patient was a woman of faith and that supporting her spiritual needs would be crucial to supporting her overall health. By recognizing that faith was deeply important to the patient, the SN had a better understanding of who the patient was and what guided her decision making. It also allowed the SN, who was also a woman of faith, to quickly connect with the patient and create trust, which was crucial to ensuring a good SN-patient relationship.

## Discussion

As the United States population ages, comprehensive programs like Henry Ford Health’s SCNP may provide a solution to the various care gaps experienced by older adults. As evidenced by this patient’s story, programs like the SCNP can provide connection to community resources (such as Meals on Wheels or PACE), social support in the form of regular telephone calls, resources for patient advocacy, and support that allowed the patient to continue living independently.

The SCNP provides an opportunity for personalized interventions as opposed to a one-size-fits-all approach. In the case study provided, the patient story illustrated various unmet needs that could not have been addressed by simply sharing resources. Instead, this is a prime example of the multifaceted needs seniors face. This aligns with previous literature that found that patient navigation can meet the evolving needs of older adult patients and has value in allowing older adults to continue living independently (aging in place) (13, 22). SNs have dedicated time to provide patient education, follow-up on a continuous basis, and establish rapport that may not be feasible in a clinical setting, which adds to the unexpected social benefits of patient navigation (13, 23).

As shown in this case, even with connection to resources, patients may still deny the use of these resources (as was the case with the PACE program). It is unclear why the patient decided to forego enrollment in the PACE program; however, initial hesitancy was also identified in previous research as an emergent theme regarding patient’s experiences with social support services (16, 24). Consequently, the patient’s SN had to compensate for this by providing similar but separate services, creating extra work for the SN. This demonstrates the challenges SNs may face when respecting each patient’s decision.

The SCNP is not without limitations. The main limitation currently is the low number of SN staff and location-based access to the SCNP. Currently the program is at 8 clinics with 2 established SNs. With 251 people having participated in the program thus far and population estimates indicating that this need will be greater in the future, expansion to other clinics must also include hiring more staff to meet growing patient needs. This is of particular importance given that a higher case load per patient navigator can lead to poor patient care (14).

There is also a fundamental link between available funding and the effectiveness of PNPs (14). Adequate resources are crucial for PNPs and there is agreement (14) that patient navigators can increase patient satisfaction, improve medication adherence, or lead to decreased no-shows or appointment cancellations; thus, more work should be done that can further evaluate similar PNPs.

The perceived effectiveness of HFH’s SCNP is limited because currently only under a third of participants agreed to completing surveys regarding their satisfaction with the program. Though this limits the generalizability of this work, answers gathered from the survey respondents provide an opportunity for the program’s improvement in the future.

## Conclusion

Older adults often struggle to care for themselves and maintain their independence due to increasing health needs as they age, and difficulty navigating complex health and social care systems. HFH’s SCNP plays a crucial role in supporting older adult patients identify and access health and social care resources and services. We hope that the description of the SCNP and case study presented herein helps to provide an example and evidence that such programs are valuable and can create meaningful impact among older adult populations.

## Data Availability

The original contributions presented in the study are included in the article/supplementary material, further inquiries can be directed to the corresponding author.
